# Comparison between robot-assisted and manual percutaneous coronary intervention - an updated systematic review, meta-analysis, propensity-matched investigation, and trial sequential analysis

**DOI:** 10.1007/s12928-025-01131-8

**Published:** 2025-05-30

**Authors:** Paweł Łajczak, Ayesha Ayesha, Oguz Kagan Sahin, Priscilla Isabel Freeman, Mir Wajid Majeed, Bruno Branco Righetto, Ogechukwu Obi, Gabriel Jacob Moreno, Mrinal Murali Krishna, Kangwa Francis Mulenga, Emma Ann Finnegan, Meghna Joseph, Anna Łajczak, Michele Schincariol

**Affiliations:** 1 Medical University of Silesia, Katowice, Poland; 2 Shifa College of Medicine, Islamabad, Pakistan; 3 Edremit State Hospital, Balikesir, Turkey; 4 Facultad de Ciencias de la Salud, Programa: Medicina, Universidad del Tolima, Ibague, Colombia; 5 Government Medical College, Srinagar, India; 6 University Positivo, Curitiba, Brazil; 7 New York Institute of Technology College of Osteopathic Medicine, Old Westbury, NY USA; 8 Centro Universitário Unidompedro Afya, Salvador, Brazil; 9 Medical College Thiruvananthapuram, Thiruvananthapuram, India; 10 Azerbaijan Medical University, Baku, Azerbaijan; 11 School of Medicine, Trinity College Dublin, Dublin, Ireland; 12 Klinikum Fürth, Friedrich-Alexander-University Erlangen- Nürnberg, Fürth, Germany

**Keywords:** Robot, PCI, Trial sequential analysis, Meta-analysis, Radiation, Safety

## Abstract

Robotic-assistance in the percutaneous coronary intervention procedures (R-PCI) has emerged as a novel alternative to manual PCI (M-PCI). However, previous reviews have not incorporated advancements in new devices. Therefore, we aim to present updated results for a comprehensive systematic review and meta-analysis comparing these two modalities.We systematically searched five databases. Clinical studies comparing R-PCI to M-PCI were included. Continuous outcomes were analyzed using a mean difference (MD), while binary outcomes were assessed with odds ratios (ORs) using random-effect models due to anticipated heterogeneity. A total of 10 papers were included. Clinical success for < 20% residual stenosis was higher (OR 7.93 (95% CI 1.02 to 61.68)), while air kerma was lower (MD − 468.61 (95% CI − 718.32 to − 218.90)) in R-PCI procedures. However, procedural time (MD 5.57 (95% CI − 5.69 to 16.84)), fluoroscopy time (MD − 0.30 (95% CI − 2.26 to 1.66)), contrast dose (MD − 6.29 (95% CI − 25.23 to 12.65)), dose area product (MD − 642.57 (95% CI − 2434.20 to 1149.07)), MACE events (OR 0.54 (95% CI 0.15 to 1.96)), and mortality (OR 1.86 (95% CI 0.82 to 4.22)) showed no significant difference between interventions. TSA showed true positive result. Our meta-analysis reveals decreased air kerma in robotic versus manual PCI but fewer statistically significant outcomes overall. Results from this study offer a more comprehensive view of existing evidence compared to previous analyses.

## Background

Robotic percutaneous coronary intervention (R-PCI) was recently introduced into the field of cardiac interventions to improve the accuracy of coronary revascularization procedures and the safety of patients and staff in the operating room [1–3]. With the use of R-PCI systems, such as CorPath 200 or CorPath GRX, this computer-aided technology can be used for remote manipulation of the catheter to perform stenting, balloon angioplasty, and more. Because this navigation can be done remotely, for example, in a shielded room, this technology allows for the reduction of dangerous fluoroscopy radiation, reducing the risk of neoplasia [4, 5]. In addition, in the shielded room, there is no need for equipping radiation shielding, minimizing musculoskeletal degeneration, which is associated with prolonged procedures in the catheter laboratory [6]. R-PCI continues to grow in numbers, and it is being widely recognized for the aforementioned benefits, showing an alternative to traditional manual PCI (M-PCI).

Traditional M-PCI has been a standard treatment for patients with coronary artery disease (CAD) for a long time. M-PCI involves the use of fluoroscopy imaging for navigation and guidance of catheter interventions, as well as balloon angioplasty and coronary stenting procedures. While M-PCI has been proven to be a successful procedure in the literature for CAD revascularization procedures, the risk of ionizing radiation to the operator and interventional staff poses a risk of neoplasm development [7]. Heavy lead protection and the repetitive nature of M-PCI may lead to significant physical strain and further musculoskeletal degeneration of the operators.

While previous meta-analyses have already compared these two procedures, these papers do not provide up-to-date analysis, as this field is rapidly developing, and technological improvements are on the way [8–10]. The aim of this review is to assess clinical endpoints and explore the technological advantages of robotic versus M-PCI techniques. This paper will incorporate the latest innovations, especially the second generation of the CorPath GRX robot model, to provide the most recent analysis. Moreover, not only do we plan to update the current literature, but we also offer a comprehensive propensity-matched analysis and trial sequential analysis, which were not performed in previous works. By this, we aim to provide more advanced techniques to explore heterogeneity and minimize potential biases.

## Methods

The Cochrane Collaboration Handbook for Systematic Reviews of Interventions and the Preferred Reporting Items for Systematic Reviews and Meta-Analysis (PRISMA) Statement Guidelines were followed for methodology and reporting [11, 12]. The protocol for this review was prospectively registered in the International Prospective Register of Systematic Reviews (PROSPERO) under the registration number (PROSPERO ID CRD42024576001).

### Eligibility criteria

Inclusion in this meta-analysis was restricted to studies that met the following eligibility criteria: (i) randomized controlled trials (RCTs) and observational studies (ii) comparing the effectiveness of percutaneous intervention (PCI) (iii) between robotic-assisted (RA) and traditional methods. Studies with institution overlap and those without a comparison group were excluded.

### Search strategy and data collection.

A comprehensive literature search of PubMed/MEDLINE, Scopus, Web of Science, Embase, and Cochrane Library, including the search terms: robot, robotically, coronary, angioplasty, PCI, stenting, revascularization, PTCA, and more—see Appendix for details. Articles were searched up to August 2024 since inception from each database. Before the final analysis, a rerun of the search was performed.

References identified from database searches were exported to Covidence. After duplicate removal, two authors independently reviewed all titles and abstracts. When discrepancies arose between the opinions of the two reviewers, a third author resolved any conflicts. All retrieved manuscripts that met the inclusion criteria were included for full-text review, with any disagreements solved by consensus.

Two independent reviewers extracted data based on titles, abstracts, and full-text articles. The data collected was organized in an electronic spreadsheet. In the case of arising conflicts, a third author was involved to make a final decision.

### Outcomes analyzed

Clinical measures were reported as provided by the individual studies. Outcomes included the clinical success rate of PCIs, time of PCI procedure, dose (volume) of contrast, time needed for fluoroscopy imaging, radiation air kerma, dose area product, 1-year mortality, and incidence of in-hospital major adverse cardiac events (MACE). Manual conversions and assistance were analyzed in the R-PCI group. Subgroup analyses were conducted based on whether studies applied propensity score matching (matched) or not (unmatched).

### Risk of bias assessment

The Risk Of Bias In Non-randomized Studies of Interventions (ROBINS-I) tool was used to evaluate bias [13]. Seven domains were assessed, including confounding, selection bias, bias in measurement classification of interventions, bias due to deviations from intended interventions, bias due to missing data, bias in measurement of outcomes, and bias in selection of the reported result. Each domain was assessed as low, moderate, serious, or critical risk. This process was performed by two authors independently. In case of disagreements, third and senior authors resolved conflicts by mutual agreement. The Robvis tool was used to display quality assessment results in plot form [14]. Certainty of evidence was assessed with GRADE [15].

### Statistical analysis

Odds ratios (ORs) with 95% confidence intervals (CIs) were employed to assess treatment effects on binary outcomes, with a p value of less than 0.05 considered indicative of statistical significance. The analysis utilized the Mantel–Haenszel (MH) test within a random-effects model to account for outcome-specific heterogeneity. Mean difference (MD) was used to compare continuous endpoints, with the Inverse Variance (IV) random-effects model. Results were illustrated using forest plots. Heterogeneity among studies was assessed using the Cochran Q test and the *I*^2^ statistic, based on Cochrane’s predefined thresholds: 0–40% indicating low heterogeneity, 30–60% moderate, 50–90% substantial, and 75–100% indicating considerable heterogeneity. Sensitivity analyses included subgroup analyses based on propensity score matching, leave-one-out sensitivity analysis, Baujat plot, and diagnostic influence.

Statistical analyses were conducted using R software 4.3.2 (The R Project for Statistical Computing, Vienna, Austria). Statistical packages included meta, metafor, ggplot2, and dmetar packages [16–19]. Conversions from median to mean were performed with the Luo and Wan methods [20, 21].

Trial sequential analysis was performed with TSA software 0.9.5.10 (The Copenhagen Trial Unit, Denmark). A random-effects model with a 95% CI was used. A two-sided 5% type 1 error was set. We used the alpha-spending O’Brien-Fleming function, with the inner wedge, empirical required information size, 80% power, and model variance-based heterogeneity correction.

## Results

**Fig. 1:**
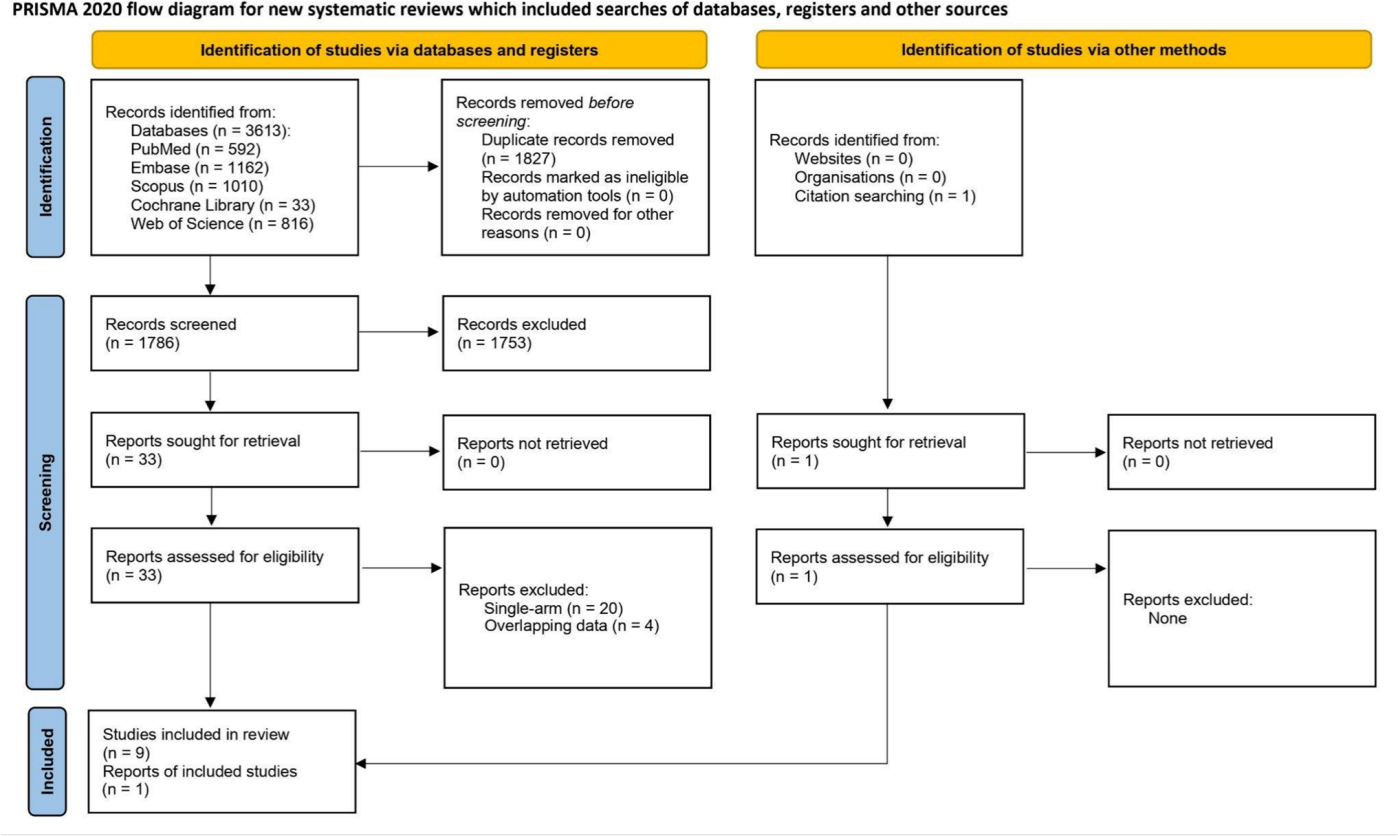
PRISMA flow diagram

The systematic search yielded 3613 results: 592 from PubMed, 1162 from Embase, 1010 from Scopus, 33 from Cochrane Library, and 816 from Web of Science. Before the screening process, 1827 duplicate papers detected by software were manually verified for similarity and removed. Title and abstract screening was performed on the remaining 1786 records. Full-text screening was performed on 33 eligible articles. We excluded 20 single-arm studies (case reports and case series) and 4 studies with overlapping data. In addition, one article was found in a manual search. Finally, ten papers, including 9 studies and 1 study report, were included in this synthesis [22–31]. Details regarding screening process are available in the Fig. 1.

A total of ten papers were included. There were no randomized studies, although six papers applied propensity matching. Most of the studies (5) used the CorPath GRX robotic system, 4 used CorPath 200, and 1 used CAAS II RNS. Mean age, number of female patients, hypertension, diabetes mellitus, myocardial infarction history, hyperlipidemia, PCI history, CABG history, smoking status, SYNTAX score, and follow-up time were collected from included studies and presented in Table 1.

**Table 1:** Baseline characteristics of included studies

Author & year	Robot	Population size (R/T)	Study design	Age (years) (R/T)	Female,n (%) (R/T)	HTN (R/T)	Diabetes Mellitus (R/T)
Bay 2023	CorPath GRX	70/210	Prospective matched- cohort	71.5 (60.2–79.8)/70.6 (62.6–77.8) #	19 (27.1)/50 (23.8)	61 (87.1)/184 (87.6)	27 (38.6)/72 (34.3)
Beyar 2006	CAAS II RNS	18/20	Clinical Trial	55.9 ± 12.0*/N/A	3(16)/N/A	(39)/N/A	(22)/NA
Hirai 2019	CorPath GRX	49/46	Retrospective observational	65.5 ± 9.2*	N/A	N/A	21(42.9)/20 (43.5)
Kagiyama 2021	CorPath GRX	30/77	Retrospective observational	70.9 ± 9.9/73.0 ± 11.3*	N/A	21(75)/63 (86.3)	17 (60.7)/39 (53.4)
Madder 2017	CorPath 200	45/291	Prospective observational	N/A	N/A	N/A	N/A
Mahmud 2017	CorPath 200	108/226	Prospective Observational	68 ± 11/67 ± 12*	24 (22)/50 (22)	102 (95)/215 (95)	61 (56)/122 (54)
Mühlen 2024	CorPath GRX	70/70	Prospective comparative	69.5 (63.0–80.0)/77.0 (68.0–82.0) #	N/A	50 (71.4)/51 (72.9)	30 (42.9)/25 (35.7)
Patel 2022	CorPath GRX-2	546/1654	Prospective observational	58 (51–65)/60 (54–68) #	122(22)/347 (21)	320 (59)/1021 (62)	237 (43)/751 (45)
Smilowitz 2014	CorPath 200	40/80	Prospective observational	64.4 ± 9.5/67.4 ± 10.5*	13 (35.6)/27 (33.7)	35 (87.5)/74(92.5)	16(40)/35 (43.8)
Walters 2018	CorPath 200	103/210	ProspectiveSingle-center observational	68.1 ± 11.0/67.5 ± 12.1*	N/A	98.3(95.4)/199.7(95.1)	57.7(56)/113.4(54)

Author & year	MI history (R/T)	Hyperlipidemia (R/T)	PCI History (R/T)	CABG History (R/T)	Smoker (R/T)	SYNTAX score (R/T)	Follow-up
Bay 2023	29 (41.4)/76 (36.2)	N.A	41 (58.6)/103 (49.0)	10 (14.3)/30 (14.3)	14 (20.0)/38 (18.1)	11.5 (7.0–18.6)/12.5 (7.0–19.8) #	12 months
Beyar 2006	(39)/NA	(83)/N/A	N/A	N/A	N/A	N/A	30 days
Hirai 2019	N/A	N/A	21 (42.9)/30 (65.2)	10 (20.4)/20 (43.5)	N/A	N/A	N/A
Kagiyama 2021	8 (28.6)/12 (16.4)	19 (67.9)/56 (76.7)	16 (57.1)/32 (43.8)	3 (10.7)/7 (9.6)	15 (53.6)/31 (42.5)	N/A	N/A
Madder 2017	N/A	N/A	N/A	N/A	N/A	N/A	30 weeks
Mahmud 2017	N/A	105 (97)/213 (94)	N/A	12 (11)/34 (15)	N/A	19.6 ± 13.0/15.7 ± 10.9*	N/A
Mühlen 2024	18 (25.7)/27 (38.6)	N/A	42 (60.0)/43 (61.4)	2 (2.9)/5 (7.1)	32 (45.7)/19 (27.1)	6.0 (4.0–8.0)/5.0 (3.0–7.0)#	12 months
Patel 2022	N/A	N/A	N/A	23 (5)/38 (2)	N/A	8 (6–13)/11 (8–18) #	N/A
Smilowitz 2014	16(40)/22 (27.5)	N/A	14(35)/30 (37.5)	5 (12.5)/6 (7.5)	24(60)/48 (60)	N/A	N/A
Walters 2018	N/A	N/A	N/A	11.3(11)/31.5(15)	N/A	19.6 ± 13.0/15.7 ± 10.9*	6–12-month

Categorical variables are expressed as n (%);*Mean ± Standard Deviation, #Median (Interquartile Range), N/A:No Information Available, R:Robotic-assisted percutaneous coronary intervention, T:Traditional methods of percutaneous coronary intervention, HTN: Hypertension, MI:Myocardial infarction, PCI: percutaneous coronary intervention, CABG:coronary artery bypass grafting

### Quality assessment

**Fig. 2:**
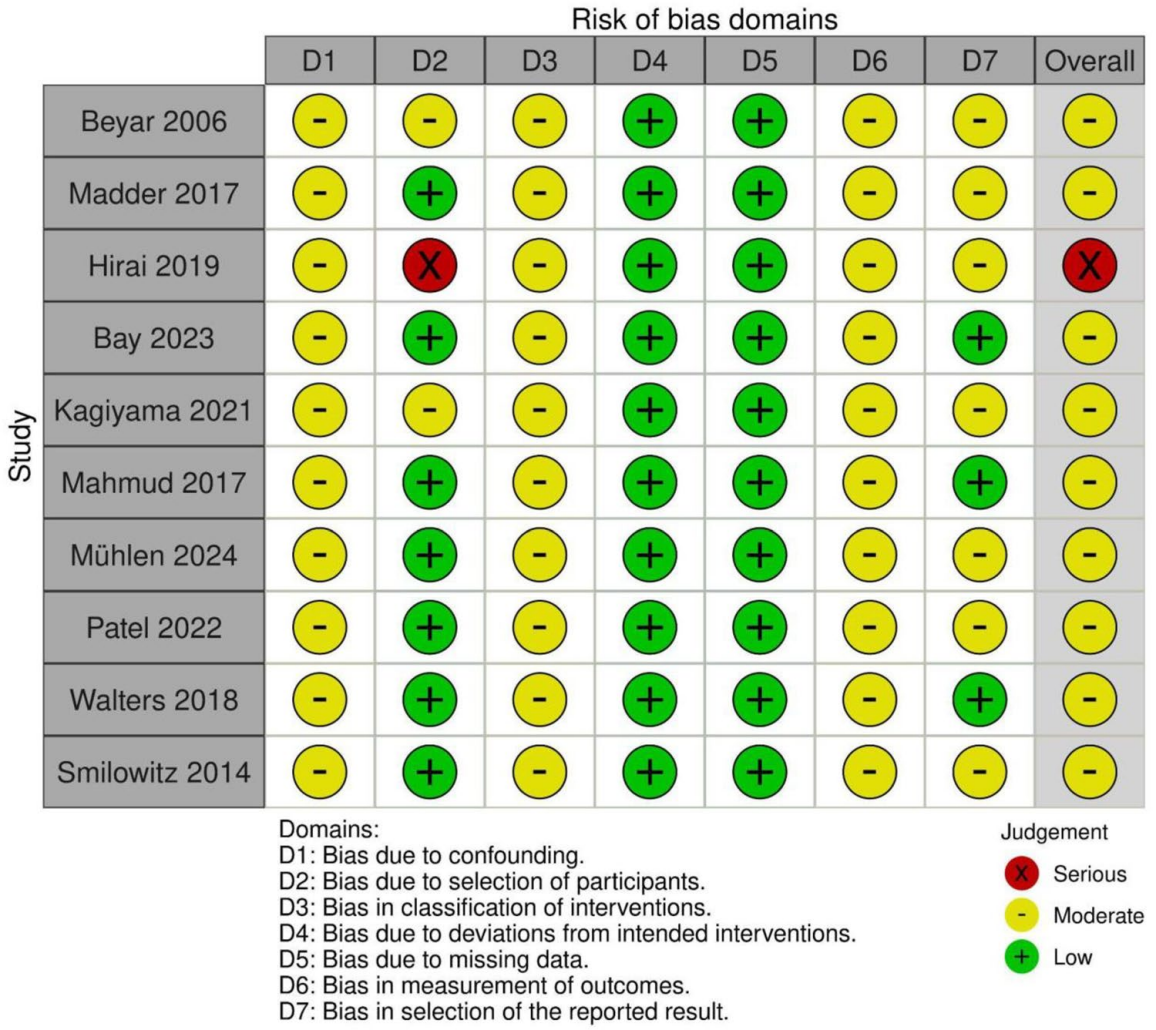
Quality assessment. Risk of bias was performed and assessed in seven ROBINS-I domains. No low-risk study was found

The risk of bias was assessed in seven ROBINS-I domains. Nearly all studies were assessed with some concerns. One study (Hirai 2019) was assessed with a high risk of bias. Most of the concerns were found in confounding, classification of interventions, and measurement of outcomes. Domains 2 and 7 had heterogeneous grading. In domains 4 and 5, low concerns were found. Figure 2 summarizes the quality assessment of the included studies.

### Meta-analysis results

#### Clinical success

**Fig. 3:**
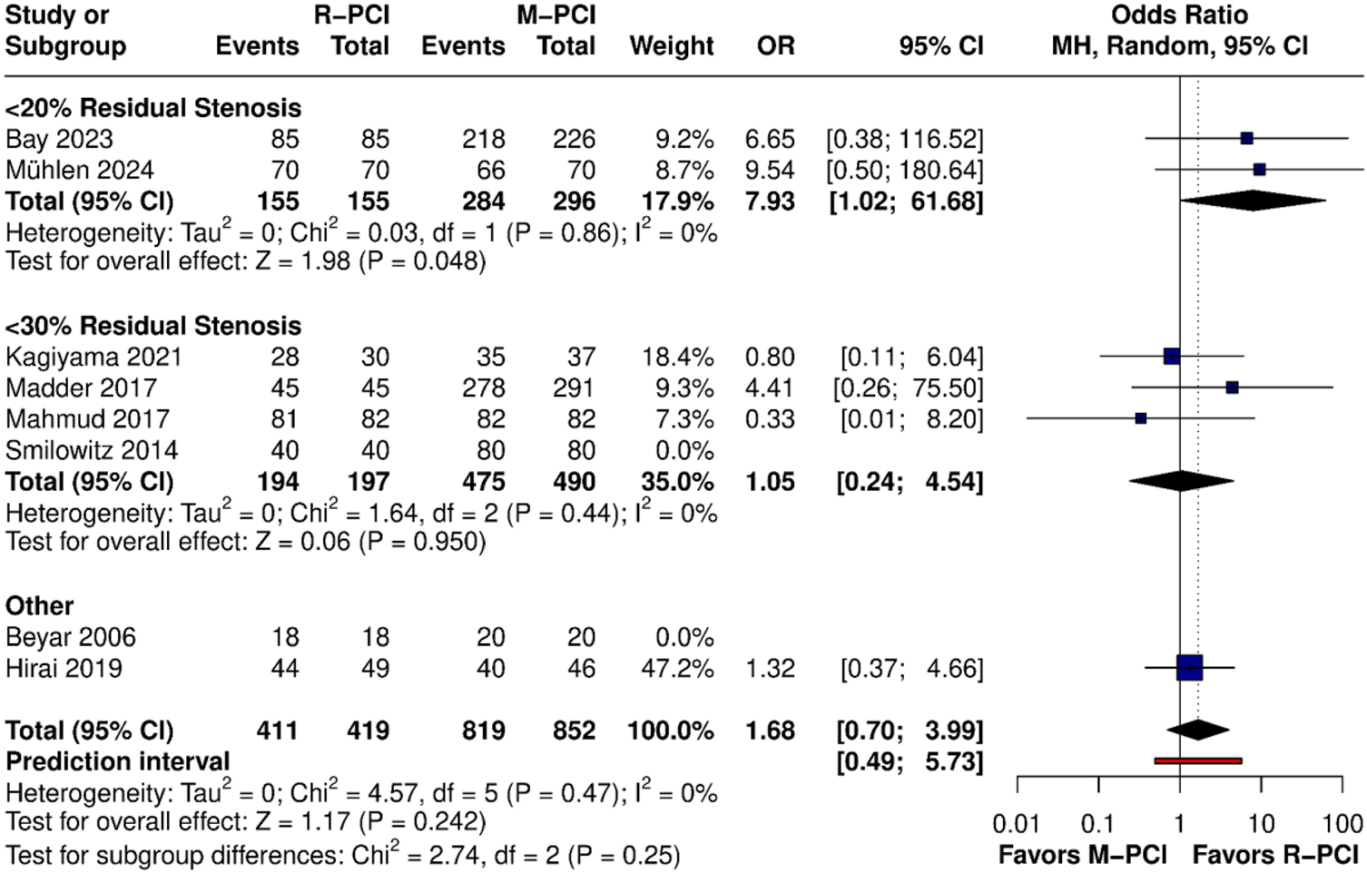
Clinical success rate forest plot

Eight studies reported clinical success. However, notable differences in the definition were observed; therefore, we performed a subgroup analysis based on it. Most studies used < 30% residual stenosis, two papers used < 20% residual stenosis, and two other papers defined clinical success based on complications or MACE (Fig. 3).

No difference was found in the overall result between R-PCI and M-PCI (OR 1.68 (95% CI 0.70 to 3.99), *p* = 0.242). No heterogeneity was observed (*I*^2^ = 0%, *p* = 0.47). However, in < 20% residual stenosis, R-PCI showed higher clinical success compared to M-PCI (OR 7.93 (95% CI 1.02 to 61.68), *p* = 0.048). Other subgroups, including propensity score-matched studies, showed no difference between R-PCI and M-PCI.

Effect size remained statistically insignificant after leave-one-out sensitivity analysis. With the Baujat plot and diagnostic influence, we were not able to find one outlier.

#### Procedure time (minutes)

**Fig. 4:**
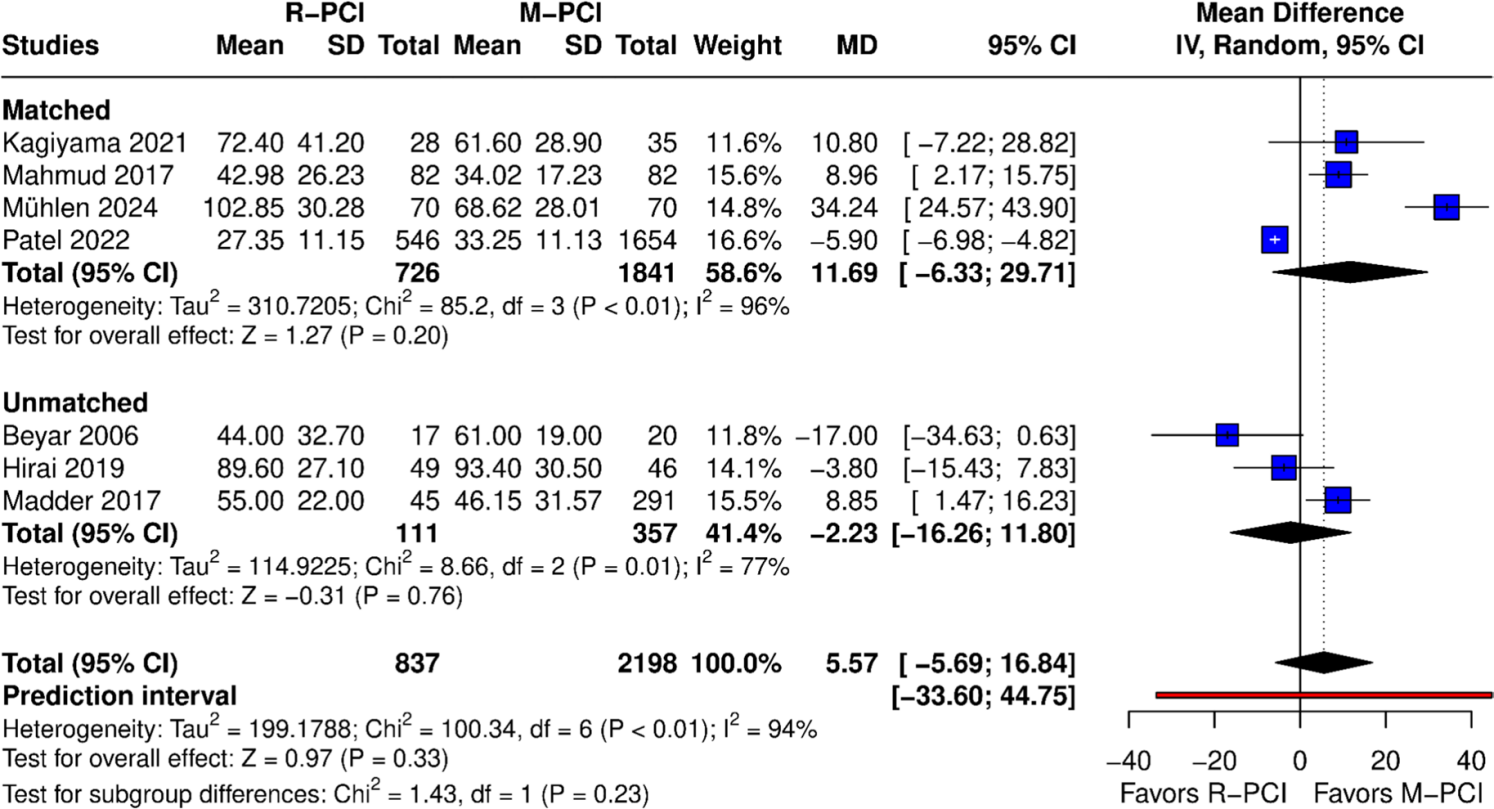
Procedure time

Procedure time was reported in 7 papers (Fig. 4). No difference was found in the overall result between R-PCI and M-PCI (MD 5.57 (95% CI − 5.69 to 16.84), *p* = 0.33). High heterogeneity was observed (*I*^2^ = 94%, *p* < 0.01). Both matched and non-matched subgroups showed no difference. Leave-one-out analysis did not influence outcome results, although the Muhlen study was detected as an outlier in the Baujat plot and influenced diagnostics.

#### Fluoroscopy time (minutes)

**Fig. 5:**
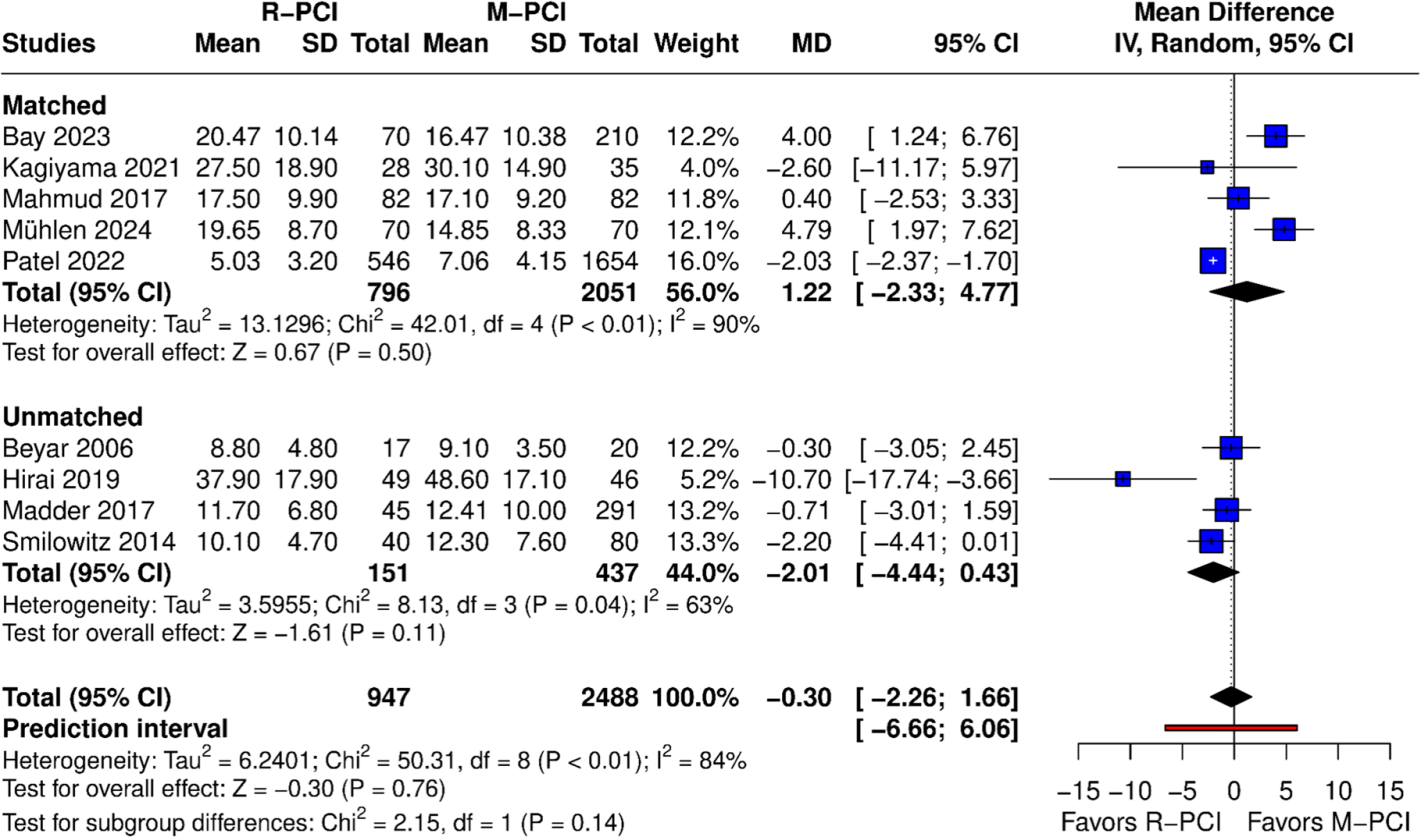
Fluoroscopy time

**Fig. 6:**
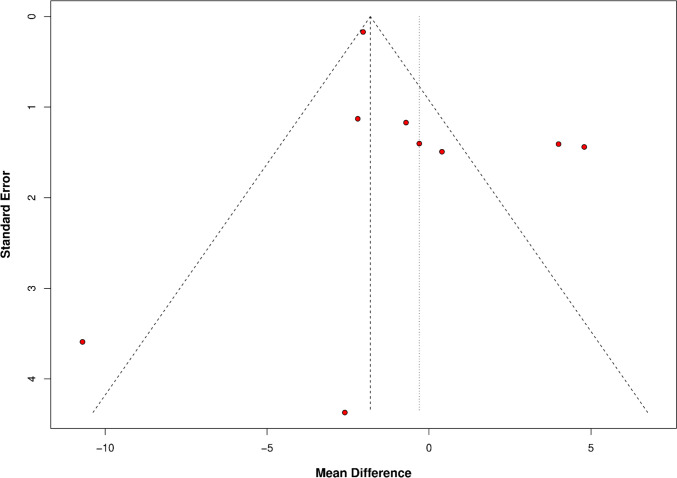
Publication bias (funnel plot)

Fluoroscopy time was reported in 9 papers (Fig. 5). No difference was found in the overall result between R-PCI and M-PCI (MD − 0.30 (95% CI − 2.26 to 1.66), *p* = 0.76). High heterogeneity was observed (*I*^2^ = 84%, *p* < 0.01). Both matched and non-matched subgroups showed no difference; however, non-matched subgroups tended to favor R-PCI more. Leave-one-out analysis did not influence outcome results. Asymmetry of the funnel plot was observed (Fig. 6).

#### Contrast volume (mL)

**Fig. 7:**
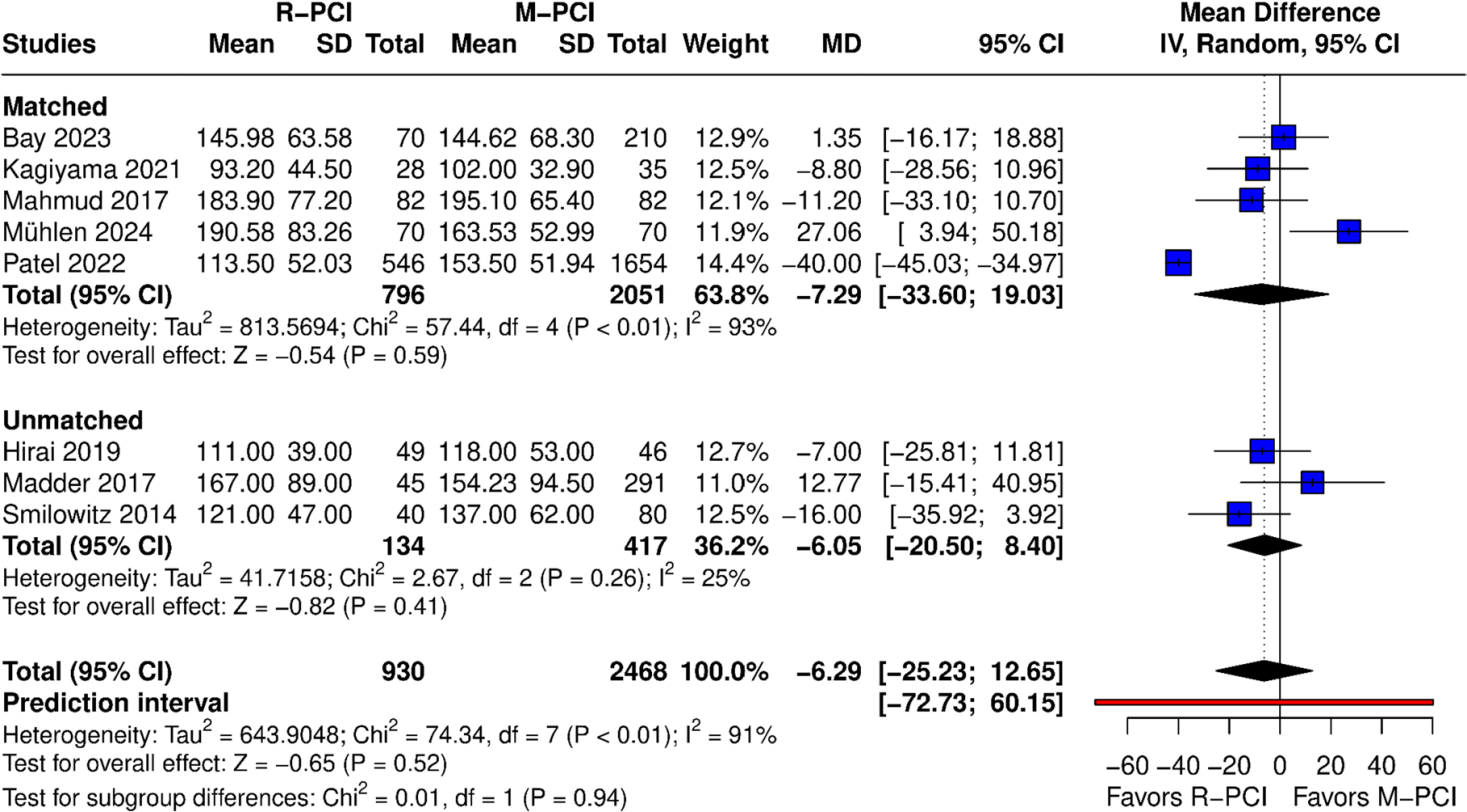
Contrast volume

Contrast volume was reported in 8 papers (Fig. 7). No difference was found in the overall result between R-PCI and M-PCI (MD − 6.29 (95% CI − 25.23 to 12.65), *p* = 0.52). High heterogeneity was observed (*I*^2^ = 91%, *p* < 0.01). Both matched and non-matched subgroups showed no difference. Leave-one-out analysis had no influence on outcome results; however, after exclusion of Patel 2022, heterogeneity dropped to 43%. This study was also identified as an outlier in influence diagnostics and the Baujat plot. The funnel plot showed asymmetry in the results.

#### Dose area product (cGycm2)

**Fig. 8:**
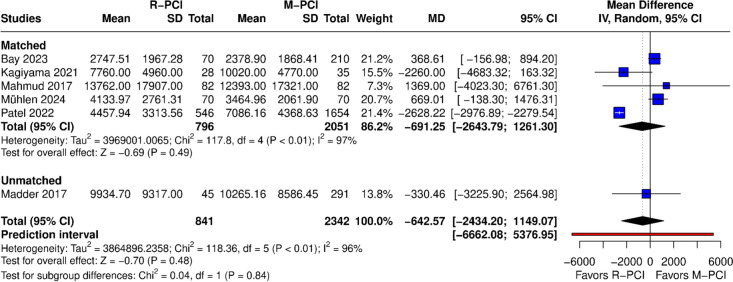
Dose area product

DAP was reported in 6 studies (Fig. 8). No difference was found in the overall result between R-PCI and M-PCI (MD − 642.57 (95% CI − 2434.20 to 1149.07), *p* = 0.48). High heterogeneity was observed (*I*^2^ = 96%, *p* < 0.01). Both matched and non-matched subgroups showed no difference.

Leave-one-out analysis had not influenced outcome results; however, after exclusion of Patel 2022, heterogeneity dropped to 26%. This study was again identified as an outlier in influence diagnostics and the Baujat plot.

#### Radiation Air Kerma (mGy)

**Fig. 9:**
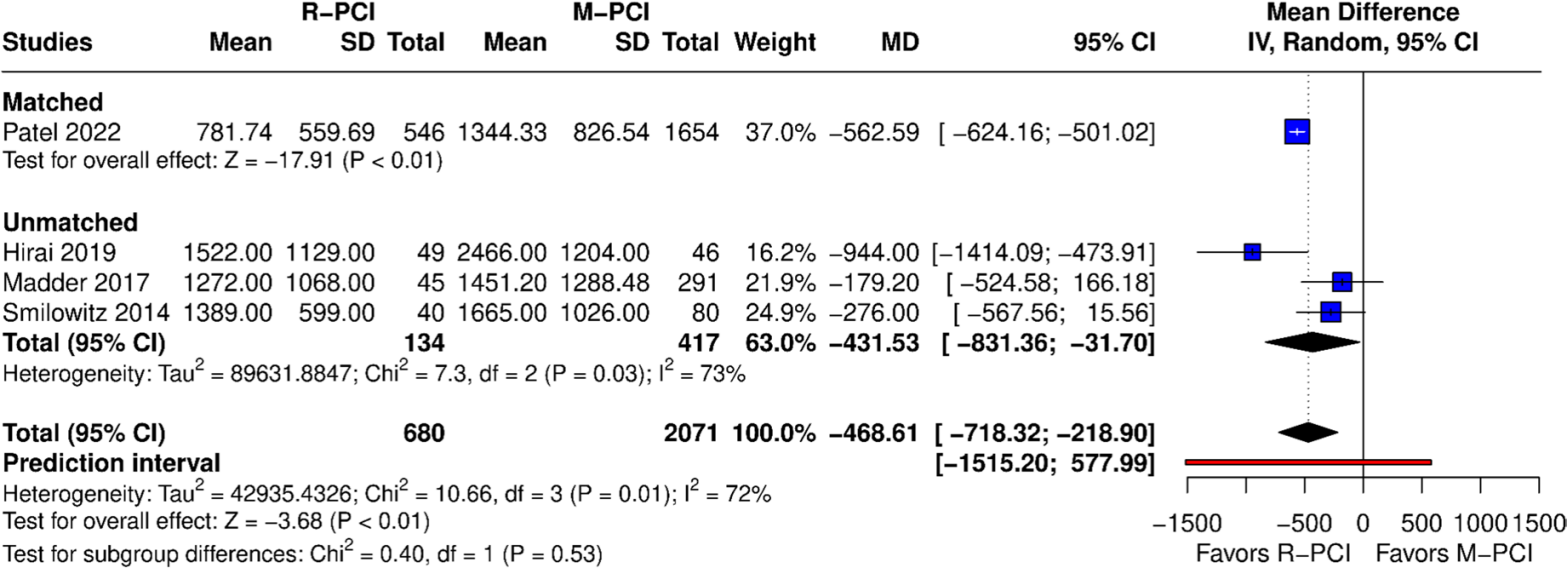
Air Kerma

Air kerma was reported in 4 studies (Fig. 9). Air kerma was lower in R-PCI when compared to M-PCI (MD − 468.61 (95% CI − 718.32 to − 218.90), *p* < 0.01). High heterogeneity was observed (*I*^2^ = 7%, *p* = 0.01). Both matched and non-matched subgroups showed lower air kerma in the R-PCI group. Leave-one-out analysis had no influence on outcome results; however, after exclusion of Patel 2022, heterogeneity dropped to 26%. This study was again identified as an outlier in influence diagnostics and the Baujat plot.

#### 1-year all-cause mortality

**Fig. 10:**
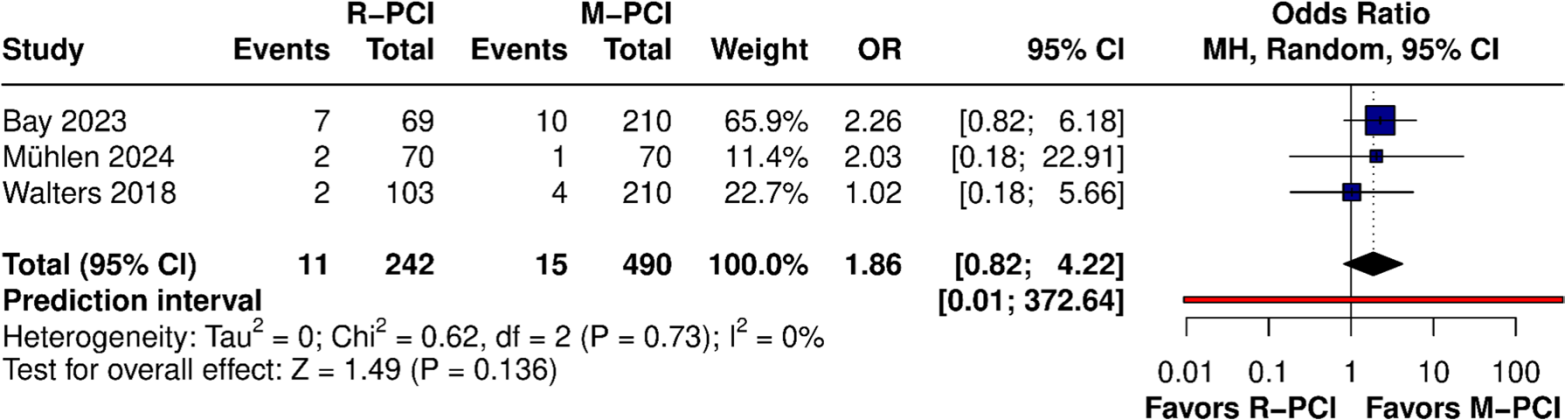
1-year ACM

1-year all-cause mortality (ACM) was reported in 3 studies, all of them matched (Fig. 10). No difference was found in the overall result between R-PCI and M-PCI (OR 1.86 (95% CI 0.82 to 4.22), *p* = 0.136). No heterogeneity was observed (*I*^2^ = 0%, *p* = 0.73). Leave-one-out analysis did not influence outcome results.

#### In-hospital MACE

**Fig. 11:**
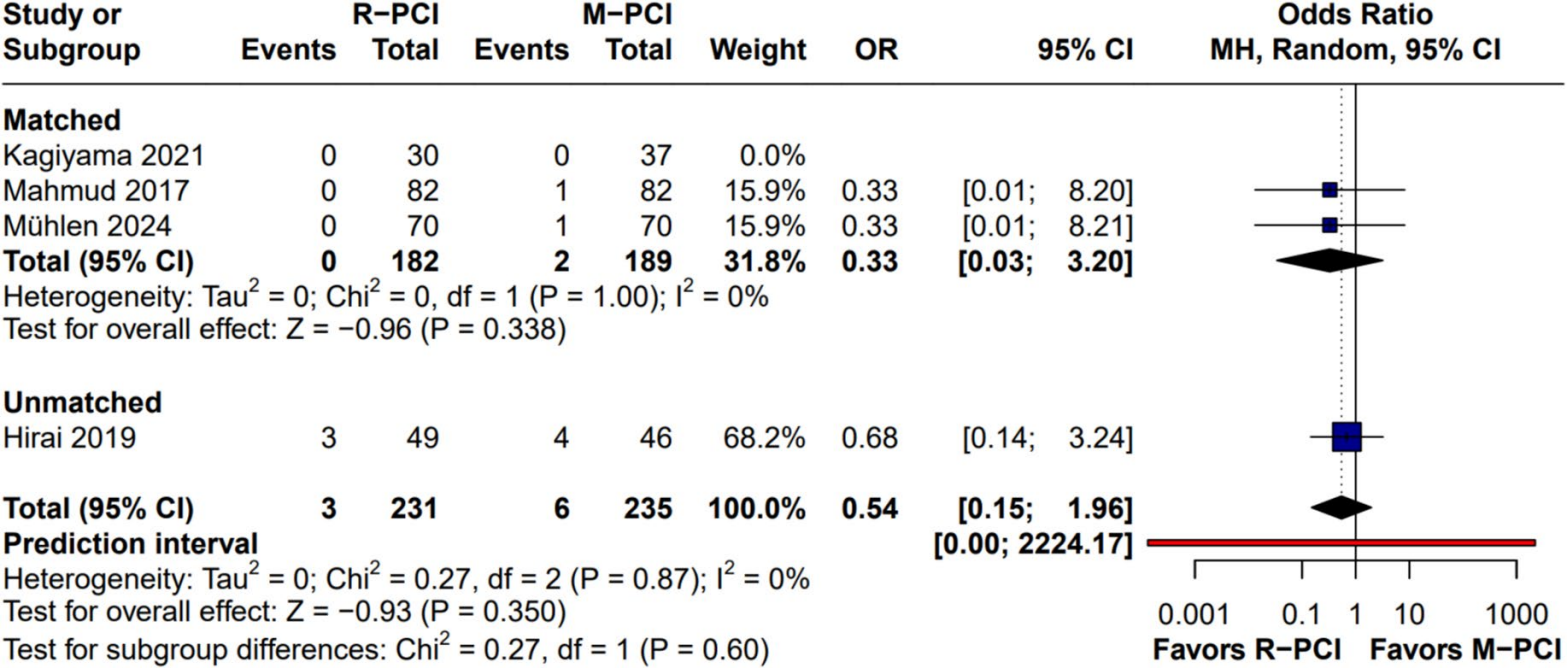
In-hospital MACE

In-hospital MACE was reported in 3 studies (Fig. 11). No difference was found in the overall result between R-PCI and M-PCI (OR 0.54 (95% CI 0.15 to 1.96), *p* = 0.35). No heterogeneity was observed (*I*^2^ = 0%, *p* = 0.87). Leave-one-out analysis did not influence outcome results.

#### Partial manual assistance

**Fig. 12:**
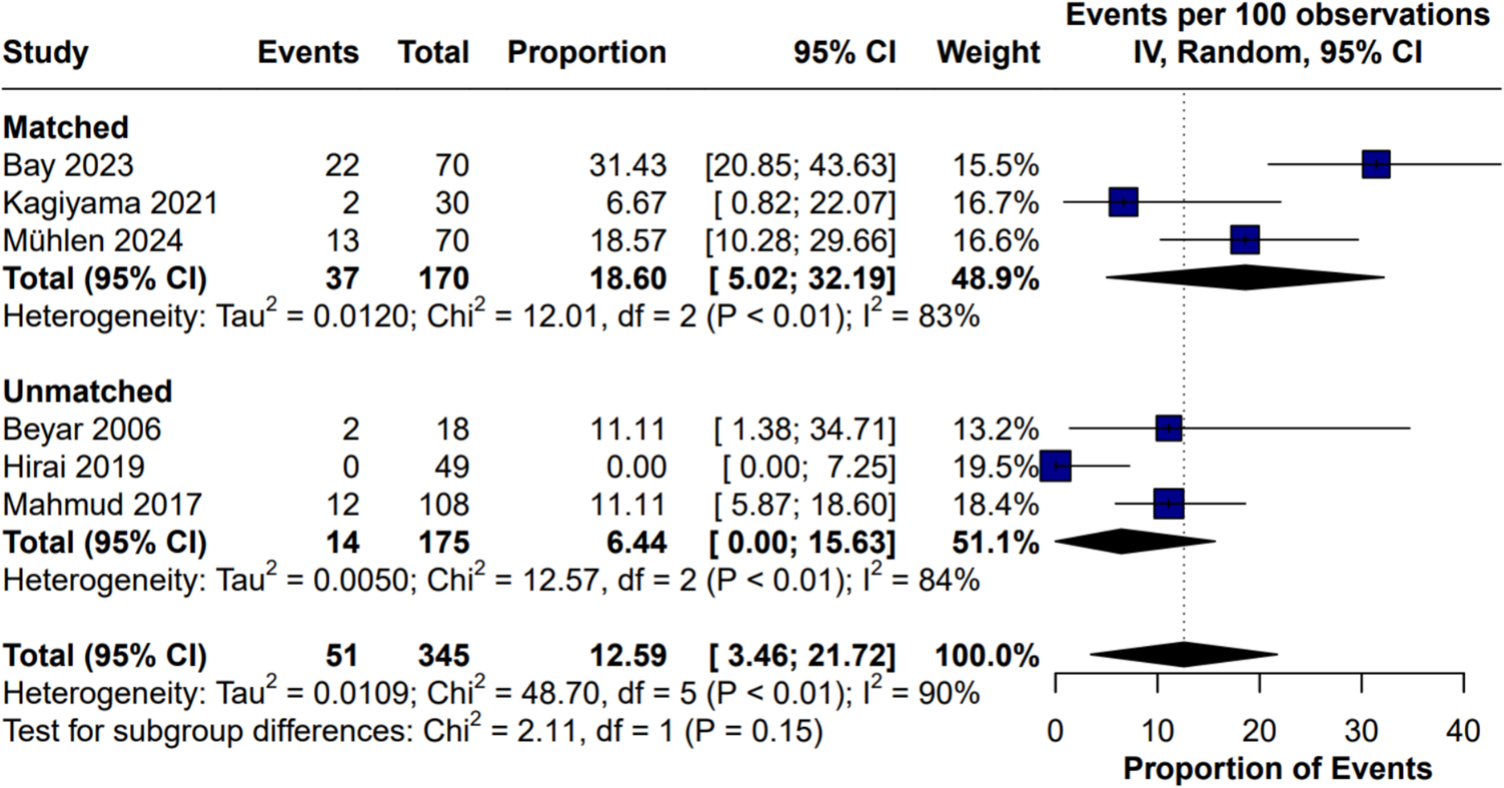
Partial manual assistance

**Fig. 13:**
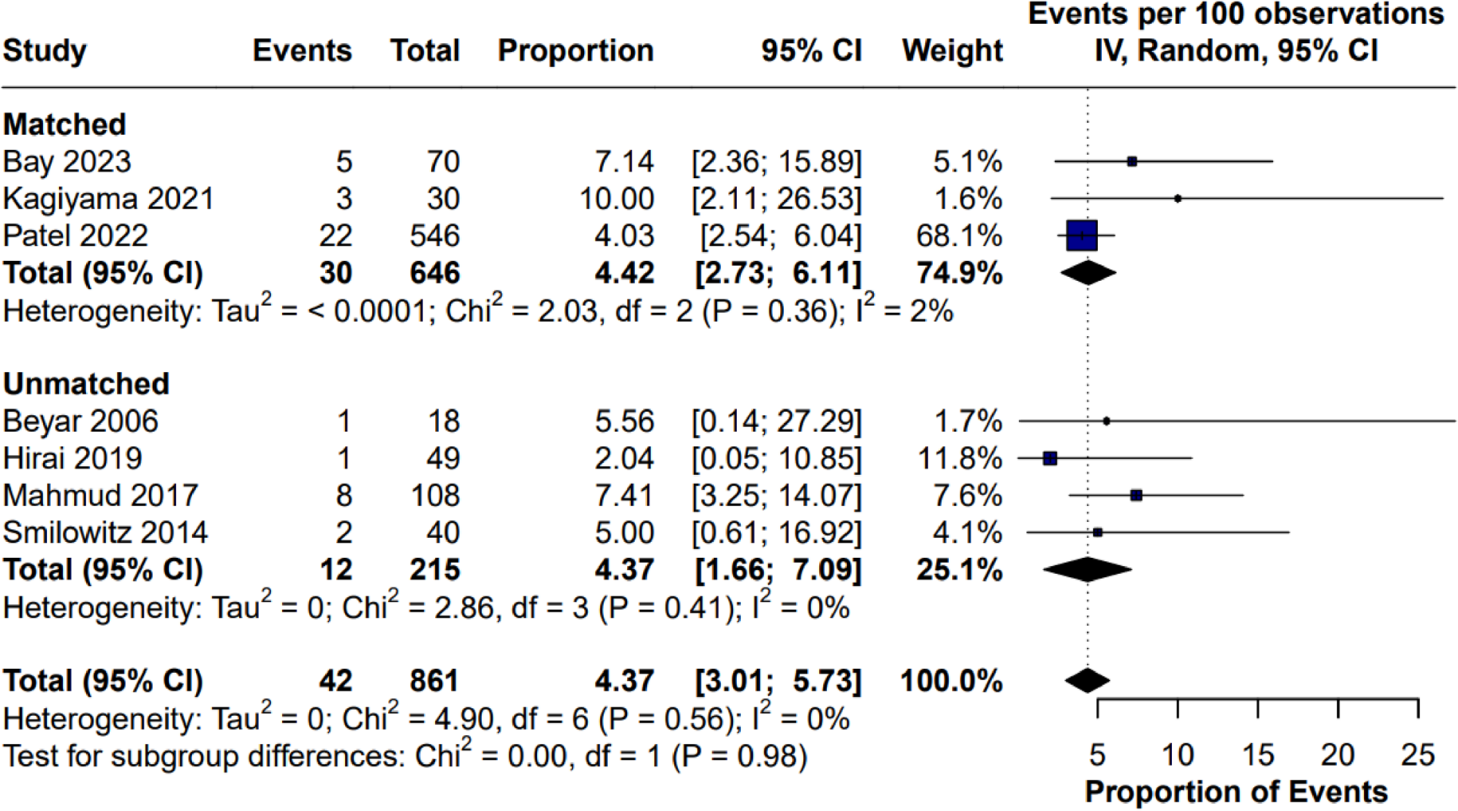
Manual conversion

Partial manual assistance was reported in 6 studies. The proportion of manual assistance was 12.59% (95% CI 3.46% to 21.72%). High heterogeneity was observed (*I*^2^ = 90%, *p* < 0.01). Leave-one-out analysis did not reduce high heterogeneity (Figs. 12, 13).

#### Manual conversion

Manual conversion was reported in 7 studies. The proportion of conversion was 4.37% (95% CI 3.01% to 5.73%). No heterogeneity was observed (*I*^2^ = 0%, *p* = 0.56).

### Trial sequential analysis

**Fig. 14:**
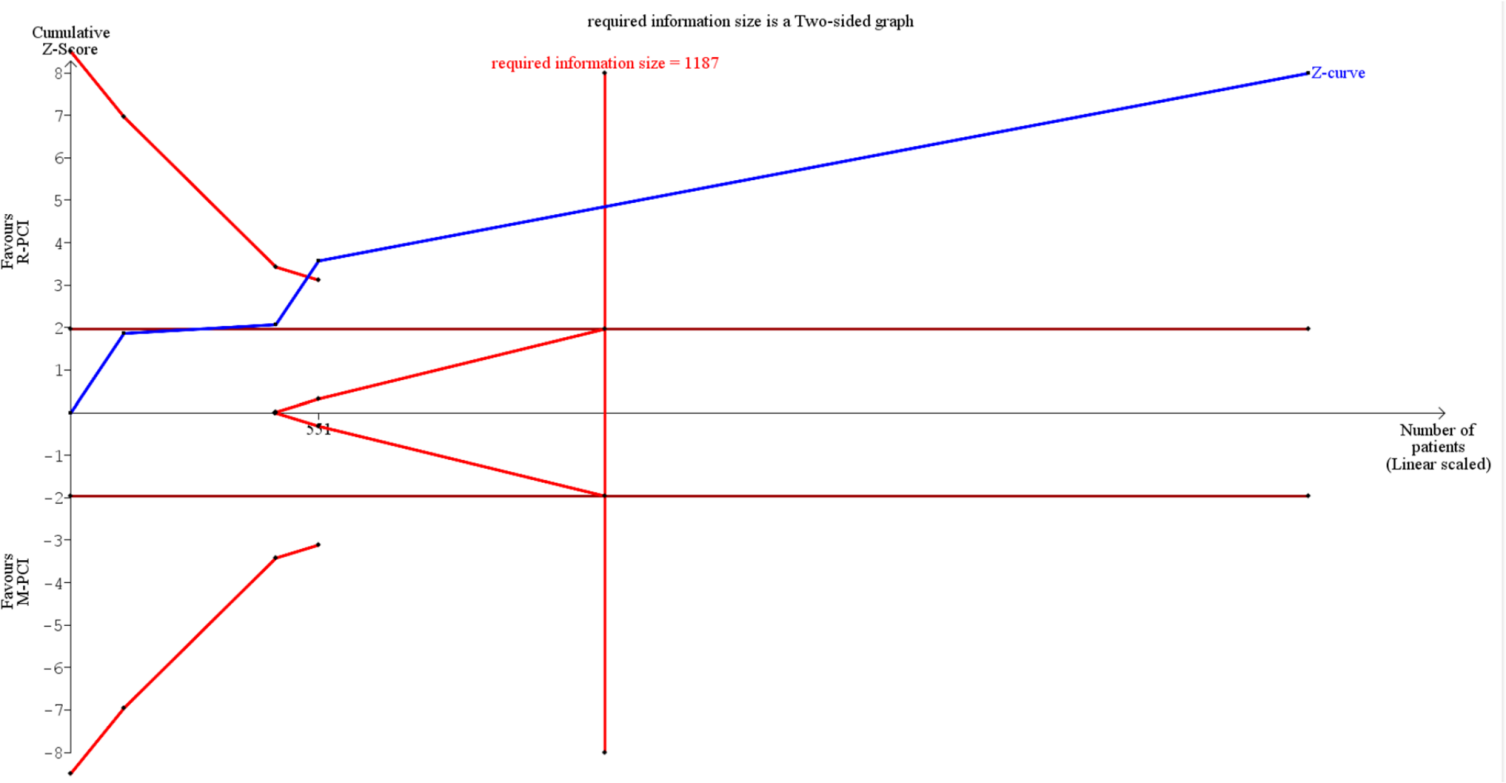
Air Kerma (TSA)

We found that Air Kerma was a true positive result, as it crossed the superiority boundary and required information size (Fig. 14).

Operation time, contrast volume, and DAP have not achieved the required patient size, and the Z-score line reached a false-negative (type 2) error.

We were unable to run fluoroscopy time TSA due to an insufficient number of patients—more than 300,000 were required for a boundary, while only 1.14% of data was available.

### GRADE certainty

Clinical success, radiation air kerma and manual conversion were graded with moderate certainty. Procedure time, fluoroscopy time, contrast volume, and DAP were graded with low certainty. A summary of the findings of the GRADE assessment is available in Table 2.

**Table 2:** GRADE assessment

Certainty assessment							No of patients		Effect		Certainty	Importance
No of studies	Study design	Risk of bias	Inconsistency	Indirectness	Imprecision	Other considerations	Robot-Assisted approach	Manual approach	Relative (95% CI)	Absolute (95% CI)		
Clinical success												
8	Non-randomized studies	Not serious	Not serious	Serious^c^	Serious^a^	Strong association	411/419 (98.1%)	819/852 (96.1%)	OR 7.93 (0.70 to 3.99)	34 more per 1,000 (from 16 fewer to 29 more)	⨁⨁⨁◯ Moderate^a,b,c^	Critical
Procedure time (minutes)												
7	Non-randomized studies	Not serious	Serious^b^	Not serious	Serious^a^	None	837	2198	–	MD 5.57 min higher (5.69 lower to 16.84 higher)	⨁⨁◯◯ Low^a,b^	Important
Fluoroscopy time (minutes)												
9	Non-randomized studies	Not serious	Serious^b^	Not serious	Serious^a^	None	947	2488	–	MD 0.3 min lower (2.26 lower to 1.66 higher)	⨁⨁◯◯ Low^a,b^	Important
Contrast volume (mL)												
8	Non-randomized studies	Not serious	Serious^b^	Not serious	Serious^a^	None	930	2468	–	MD 6.29 mL lower (25.23 lower to 12.65 higher)	⨁⨁◯◯ Low^a,b^	Important
Dose Area Product (cGycm2)												
6	Non-randomized studies	Not serious	Serious^b^	Not serious	Serious^a^	None	841	2342	–	MD 642.57 cGycm2 lower (2434.2 lower to 1149.07 higher)	⨁⨁◯◯ Low^a,b^	Important
Radiation Air Kerma (mGy)												
4	Non-randomized studies	Not serious	Serious^b^	Not serious	Not serious	None	680	2071	–	MD 468.61 mGy lower (718.32 lower to 218.9 lower)	⨁⨁⨁◯ Moderate^b^	Important
Manual conversion												
7	Non-randomized studies	Not serious	Serious^b^	Not serious	not serious	None	The proportion of conversion was 4.37% (95% CI 3.01% to 5.73%)				⨁⨁⨁◯ Moderate^b^	Important

**CI:** confidence interval; **MD:** mean difference; **OR:** odds ratio

a. wide confidence interval. b. I2 > 50%. c.there are differences in outcome definitions

## Discussion

Robots are under constant development and revolutionize modern operating rooms. This paper aimed to synthesize up-to-date analysis of robotic PCI (R-PCI) versus manual PCI (M-PCI). The results of this study demonstrate that the use of robotic assistance yields safety and clinical outcomes comparable to traditional methods, including clinical success of PCI, procedure length, fluoroscopy time, volume of contrast used, dose area product, 1-year ACM, and in-hospital MACE events [32]. Notably, the use of R-PCI led to higher clinical success rates for < 20% residual stenosis analyses and air kerma radiation.

The subgroup analysis of clinical success shows that R-PCI is significantly superior in < 20% residual stenosis. This is the only significantly superior outcome within the subgroups and should be interpreted as a successful angiographic result, considering that it is not linked to other outcomes and is therefore independent. However, as stated in Bay 2023, this finding may be the result of a selection bias, excluding patients deemed ineligible for R-PCI [22]. Nevertheless, in our meta-analysis, the risk of bias due to participant selection was found to be low.

Robot-assisted PCI is emerging as a novel tool among interventional cardiologists, offering enhanced precision through computer-assisted imaging and reduced radiation exposure for the invasive cardiology staff. The robotic platform also improves operator ergonomics during the procedure [33]. R-PCI is comparable with M-PCI, underscoring its viability as an alternative approach for revascularization [27]. This comparability in clinical safety is particularly important for clinical application, as PCI operators are often exposed to ionizing radiation, especially in high-volume catheterization labs. Such exposure to ionizing fluoroscopy radiation carries the risk of neoplasms to PCI operators.

Main clinical endpoints, including target lesion revascularization and the incidence of major adverse cardiovascular events (MACE), show no significant differences between the two methods. Both approaches can achieve comparable clinical success. R-PCI does not lead to reduced procedure times, likely due to the need for setup preparation of the robotic system, which is time-consuming. Moreover, procedure time could be influenced by the learning curve, which leads to a lack of observed differences between the two groups. The lack of these differences could be impacted by the design of robotic systems, which aim for radiation reduction, rather than significant improvement in other clinical endpoints. These outcomes could be improved in future, as technology improves. For example, the introduction of 6G networks, augmented-reality imaging, and convolutional neural network aid for the PCI itself. However, the current study confirms, that PCI is a well-established method, and most likely significant technological improvements are needed to impact its effectiveness.

However, the robotic system, thanks to its computer-assisted technology, may minimize potential hand tremors, which could lead to vessel damage and other complications for the patients [34]. Computer navigation leads to smaller navigation misses of the catheter. In addition, robotic systems are resistant to physiologic fatigue, which often occurs during prolonged procedures in the catheter lab.

The major advantage of R-PCI is the improvement of operator’s safety, due to reduction of radiation exposure during the interventions. Traditional M-PCI requires operators to perform procedures in close proximity to radiation, leading to exposure to ionizing radiation. This also necessitates the use of lead apron during interventions, which can cause musculoskeletal disorders with prolonged use. During R-PCI, the operator can navigate the catheter from the shielded console, which is often located in a separate room. This reduces not only radiation exposure but also improves operator ergonomics, being especially beneficial for interventional cardiologists, who perform multiple procedures daily.

However, while R-PCI offers various advantages for the operator, there are several limitations associated with the wide implementation of this technology in clinical practice. First, a learning curve is observed with the introduction of this technology. Operators who are adapting from M-PCI to R-PCI may require additional training to develop efficacy with computer visualization, catheter navigation, functionalities, and robotic system controls [30]. The initial stages of the learning curve may lead to slightly longer procedure time, higher reliance on M-PCI, and most likely higher odds for partial manual assistance or even full conversion to M-PCI, especially in complex revascularization cases. While the learning curve can be overcome with more experience, these aspects highlight the issue of the need for training during the initial transition period for inexperienced operators. In addition, the switch from haptic feedback present in M-PCI to visual feedback in R-PCI further makes the transition difficult [35]. The learning curve effect was analyzed in the study of Patel, which was the biggest comparative study conducted so far [29]. The authors from Apex Heart Institute in India reported that between 15 and 50 procedures were needed to achieve proficiency for R-PCI in terms of fluoroscopy time, procedure time, and dose of the contrast. Therefore, the learning curve of R-PCI is approximately 50 cases. The learning curve is caused by the fact that the operator needs to learn ergonomics and controls of the robotic workstation, while catheterization laboratory staff must learn how to prepare the robotic system and single-use disposables for the intervention. However, it is worth noting that several studies included less than 50 cases of R-PCI; therefore, the learning curve effect could impact the clinical endpoints, especially in the early stage of experience. Unlike M-PCI, where tactile feedback plays a crucial role in navigating coronary anatomy and deploying devices, robotic systems rely on visual and instrumental cues. This shift in the sensory feedback necessitates a period of adaptation for operators, particularly in scenarios requiring fine guidewire manipulation or troubleshooting in tortuous anatomies. These aspects may require even more comprehensive training and hundreds of hours with R-PCI for full adaptation.

While the learning curve is notably present at the beginning of adaptation, long-term benefits may outweigh initial challenges. With the increasing proficiency in R-PCI, the operator achieves comparable procedure time to M-PCI. Further advancements in robotic technology, which include improved haptic feedback, artificial intelligence (AI) integration, or even fully autonomous intervention, have the potential to further streamline procedures and reduce operator dependency on manual adjustments.

Furthermore, R-PCI as an alternative approach may address specific clinical scenarios where M-PCI might not be adequate for such a procedure. For example, increased precision of robotic systems could be especially advantageous in performing procedures with overlapping stents, managing bifurcation lesions, or navigating chronic total occlusions [36, 37]. Although current evidence primarily focuses on routine PCI, ongoing research and technological refinements are likely to expand the applicability of robotic systems to these complex cases.

Another important limitation of R-PCI is the high purchase cost for model acquisition, limiting availability in resource-limited settings [38]. However, such analysis could not be performed in this study. Further research should not only explore cost-efficacy compared to traditional methods but also compare quality of life after both types of procedures.

The inclusion of studies with both propensity-matched and unmatched designs introduces some between-study variability. Even though there were no randomized studies available, propensity score matching makes it possible for the two groups (R-PCI and M-PCI) to have more similar baseline characteristics. Traditional observational unmatched studies may represent under- or overestimated results due to biases in terms of confounding between the two groups. However, in the meta-analysis, we observed no significant differences between both matched and unmatched studies in clinical outcomes, including fluoroscopy time, contrast volume, or radiation air kerma. Some trend of lower fluoroscopy time was observed in the fluoroscopy outcome in the unmatched group; however, it did not reach significant results. Therefore, matching leads to less biased matching of patients in the two groups, providing more accurate comparative findings of the analysis.

This systematic review is limited by lack of randomized controlled studies, which are currently not present. Reliance on retrospective studies leads to increased bias and potential overoptimistic interpretation of the results.

We observed inconsistency in some outcomes, which could not be explained through robust sensitivity analyses. Meta-regression could not be employed, as there were < 10 studies.

## Conclusions

In conclusion, R-PCI represents a viable alternative to M-PCI, offering comparable efficacy and distinct advantages in operator safety and procedural precision. While the learning curve associated with robotic systems presents an initial challenge, targeted training and experience can mitigate these barriers, enabling operators to fully leverage the benefits of this technology. As robotic systems continue to evolve and address current limitations, their role in interventional cardiology is likely to expand, paving the way for safer, more efficient, and more precise treatments for coronary artery disease. To realize the full potential of R-PCI, ongoing investment in training, research, and technological innovation will be essential.

## Declarations

### Use of Large Language Model

Authors declare the use of Gemini LLM (Google Inc.) only for grammar revision and writing corrections. LLM was not used to generate scientific content; all changes were double-verified by the authors of the study, and the authors take full responsibility for these changes.
